# Preparation and Characterization of Self-Reinforced Antibacterial and Oil-Resistant Paper Using a NaOH/Urea/ZnO Solution

**DOI:** 10.1371/journal.pone.0140603

**Published:** 2015-10-14

**Authors:** Li Jiao, Jinxia Ma, Hongqi Dai

**Affiliations:** Department of Pulp and Paper Science and Technology, Nanjing Forestry University, Nanjing, Jiangsu, China; Institute for Materials Science, GERMANY

## Abstract

This paper describes self-reinforced antibacterial and oil-resistant properties that were successfully prepared by surface selective dissolution of filter paper in a NaOH/Urea/ZnO (weight ratio of 8:12:0.25) aqueous solution. The effect of the processing time on the mechanical properties of this paper was evaluated at -12°C. The paper morphologies were characterized using Scanning Electron Microscopy (SEM), X-ray Diffraction (XRD), Fourier Transform Infrared Spectroscopy (FT-IR) and X-ray photoelectron spectroscopy (XPS). The oil-resistance and antibacterial properties of the produced paper were also investigated. Excellent mechanical properties were observed for an optimized handling time. The tensile and burst strengths of the modified paper were in excess of 100% of the original. Meanwhile, the treated paper was completely oil-resistant within 24 h and demonstrated good antibacterial properties when exposed to *Staphylococcus aureus*. The traces of residual zinc oxide were found to be safe for food.

## Introduction

Many efforts have been undertaken to obtain sustainable, biodegradable material to replace glass, plastic and metal in packaging due to increasing environmental issues. Cellulose-based paper is considered to be an environmental friendly and cost-effective alternative packaging material because of its easy manufacturing and excellent mechanical and surface properties [1]. However, regular paper cannot prevent oil permeation and bacterial invasion due to the presence of hydroxyl groups on the fibre surfaces, which limits their usage in the packaging of fatty foods.

Cellulose oil resistance can be improved by beating the pulp, which has been extensively used in the manufacture of oil-resistance paper. However, increasing the degree of beating generally leads to dewatering, pressing and drying problems [2]. Surface coatings using synthetic polymers or natural polysaccharides are another way to enhance the oil-resistance of the surfaces of cellulose materials [3]. However, synthetic polymers are non-biodegradable and toxic due to the presence of volatile monomers in the food packaging. Due to increasing concerns regarding the environment and food safety, a decline in synthetic polymer utilization in food packaging is inevitable. The high cost of some natural polysaccharides, such as chitosan and sodium alginate, and so on, limit their application in food packing. Some proteins, including isolated soy protein (ISP), whey protein isolate (WPI) and wheat gluten can also be coated on the surface of paper to protect against oil permeation. However, poor mechanical properties are observed for paper coated by ISP and WPI and are not suitable for packaging. In addition, oil proof paper may be fabricated by laminating with aluminium foil. Unfortunately, this type of laminated paper exhibited issues, including difficult recovery and high cost. In addition to beating, coating, calendaring and laminating, surface modification by chemicals is another approach to increase the oil resistance. Theoretically, a cell wall of cellulose fibre is formed by several layers, with the outer layers being less ordered and poorly oriented, making it easy to dissolve them in some solvents. The dissolved fibers can fill in paper pores and cover un-dissolved core fibers, minimizing voids to prevent the permeation of oil [4–7].

Cellulose is regarded as an amphiphilic macromolecule [8–10]. Many researchers have noted that excellent cellulose solvents should be able to dissolve hydrogen bonds and generate hydrophobic interactions between the cellulose molecules [11,12]. Currently, several solvents are available for dissolving cellulose: lithium chloride/N, N-dimethylacetamide (LiCl/DMAc), N_2_O_4_-dimethylformamide (DMF), N-methylmorpholine-N-oxide monohydrate (NMMO) and ionic liquids, which had been reported. However, these processes are limited to laboratory scale applications due to their volatility, toxicity, and high cost. Recently, NaOH/urea systems for cotton linter dissolution have been successfully developed. Using this compound is considered to be a promising method of dissolving cellulose due its cost-effective nature and environmental friendly raw materials [13]. Some additives, such as thiourea and ZnO, can enhance the dissolution power of the system for cellulose. ZnO exists as Zn(OH)_4_^2-^ in an alkali solution, which can form stronger hydrogen bonds with cellulose than NaOH hydrate[14]. Therefore, NaOH/urea/ZnO may be a promising solvent for cellulose. However, high viscosity-molecular weight (Mη) (M_η_>14×10^4^) or high crystallinity pulp cannot be completely dissolved in this solution.

Taking advantage of the limited dissolution capacity, a cellulose solvent (8%NaOH/12% urea/0.25%ZnO by weight) was used to dissolve the surface layer of filter paper possessing less-order and poor orientation. Though compression and drying, the dissolved fibres are absorbed and covered on the highly oriented un-dissolved fibres, which is a key for the high mechanical performance of the paper. During regeneration, a significant number of hydrogen bonds were regenerated. High quality interfacial bonds were formed between the dissolved and un-dissolved fibres, which permitted good stress-transfer. Interfacial bonding using the same components can overcome the disadvantages associated with interfacial incompatibility that exist between the matrix and a reinforcement material composed of a different component in composites. The dissolved fibres provide oil-resistance and mechanical reinforcement. Recently, the preparation of all-cellulose composites using selectively dissolved cellulose fibre was reported using a matrix reinforced with core cellulose. The tensile strength of the all-cellulose composite was 211 MPa, which is higher than for a conventional glass-fiber-material reinforced composite[15–17].

**Fig 1:**
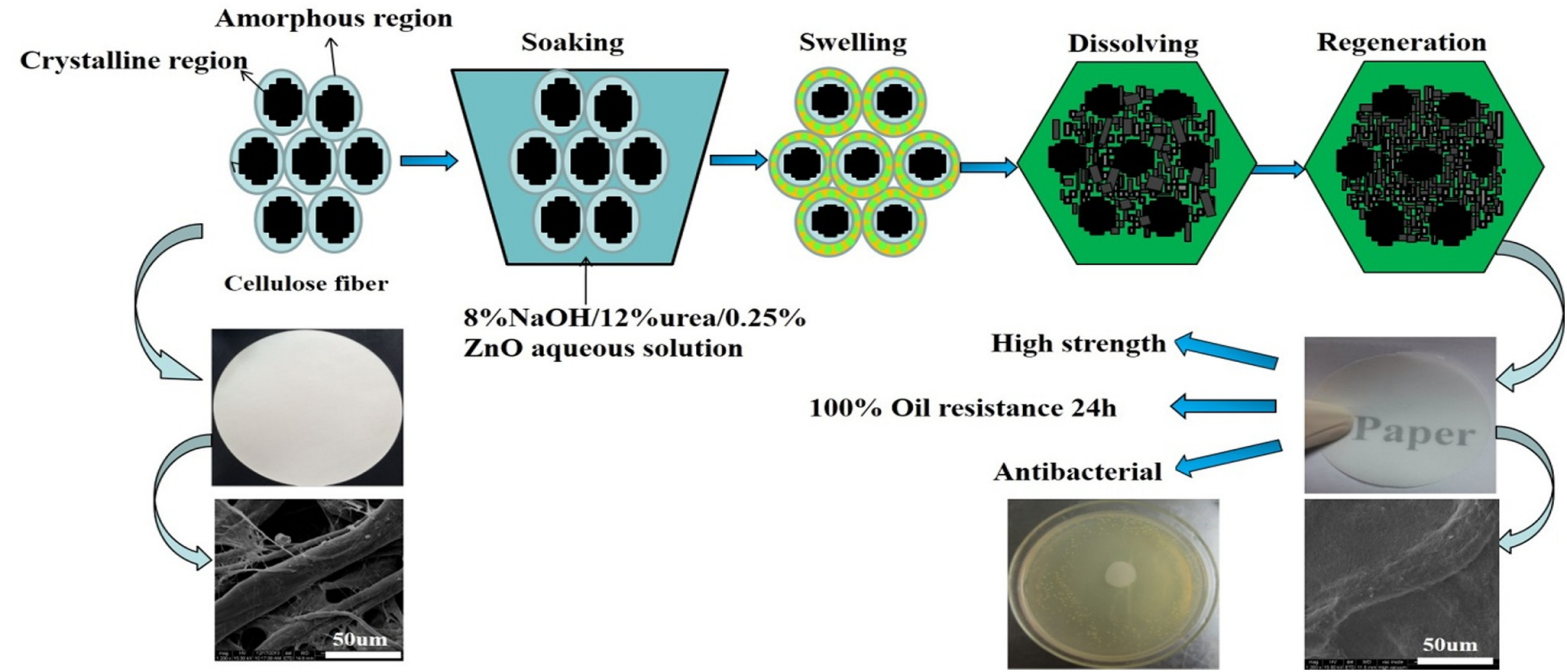
Schematic diagram of the procedure for preparing self-reinforced antibacterial and oil-resistance paper.

Some residual ZnO would provide the paper with an antibacterial property due to the efficient antibacterial properties of ZnO and would also provide strong thermal stability and durability for packaging. Organic antibacterial agents have been used to prepare antibacterial papers, but some are volatile and exhibit poor thermal stability. Additionally, some monomers emit when used for long periods of time, which limit their use in food packaging. Researchers have modified cellulose fibres with metallic salts or using surface graft polymerization to yield antibacterial papers. However, this method is not cost-effective or environment friendly [18,19]. In this study, ZnO is stably coated on the surface fibres and cell lumen and yield treated paper with an antibacterial property. Therefore, fibre paper treated using 8%NaOH/12%urea/0.25%ZnO exhibits multiple-functions, including high strength performance, oil resistance and antibacterial properties. The key objective of this work is the development an environmentally friendly technology for the production of paper with oil resistance, antibacterial properties and improved mechanical strength. The schematic diagram for preparation of functional sample can be seen in Fig 1.

## Materials and Methods

### Materials

Filter paper with a base weight of 103 g/m^2^ and a diameter of 18.5 cm was purchased from Fisher Scientific International, Inc. (Pittsburgh, UK). The paper was made from cellulose fibres with a degree of polymerization (DP) of approximately 830. Sodium hydroxide, urea and zinc oxide were purchased from Nan Jing chemical reagent Co. LTD. (Nan Jing, China). All of the chemicals were used as received.

### Sample production

The NaOH/urea/ZnO/water solution was prepared with a ratio of 8:12:0.25:79.75 (by weight). After the solution was pre-cooled to -12°C, the filter paper was impregnated into the solution for 30 seconds at 25°C to ensure good saturation. Then, the treated paper was taken out and cooled to -12°C for 30 to 180 minutes. Subsequently, the treated paper was compressed between two clear plastic sheets at 5.0 KPa for 3 minutes and rinsed several times using ultrapure water to ensure the complete removal of the NaOH and urea. Finally, the treated paper was dried in a vacuum at 100°C and 0.1 MPa for 10 minutes. All of the treated papers were kept at 25°C and 50% relative humidity for 24 h. The effects of the treating time at -12°C on the mechanical properties and morphology of the treated paper were then systematically investigated.

### Measurements

#### Mechanical properties

The tensile strength was measured in accordance with the TAPPI method T 494 om-01 using a tensile tester (WZL-300, Instrument Development Co., Hang Zhou, China) at room temperature. Burst strength testing was performed in accordance with the TAPPI method T 403 om-02 using a bursting tester (YQ-Z23A, light industrial instrument plant, Hang Zhou, China). The average value and standard deviation of the tensile and burst indexes were calculated for at least five sets of samples.

#### Oil-resistance test

**Fig 2:**
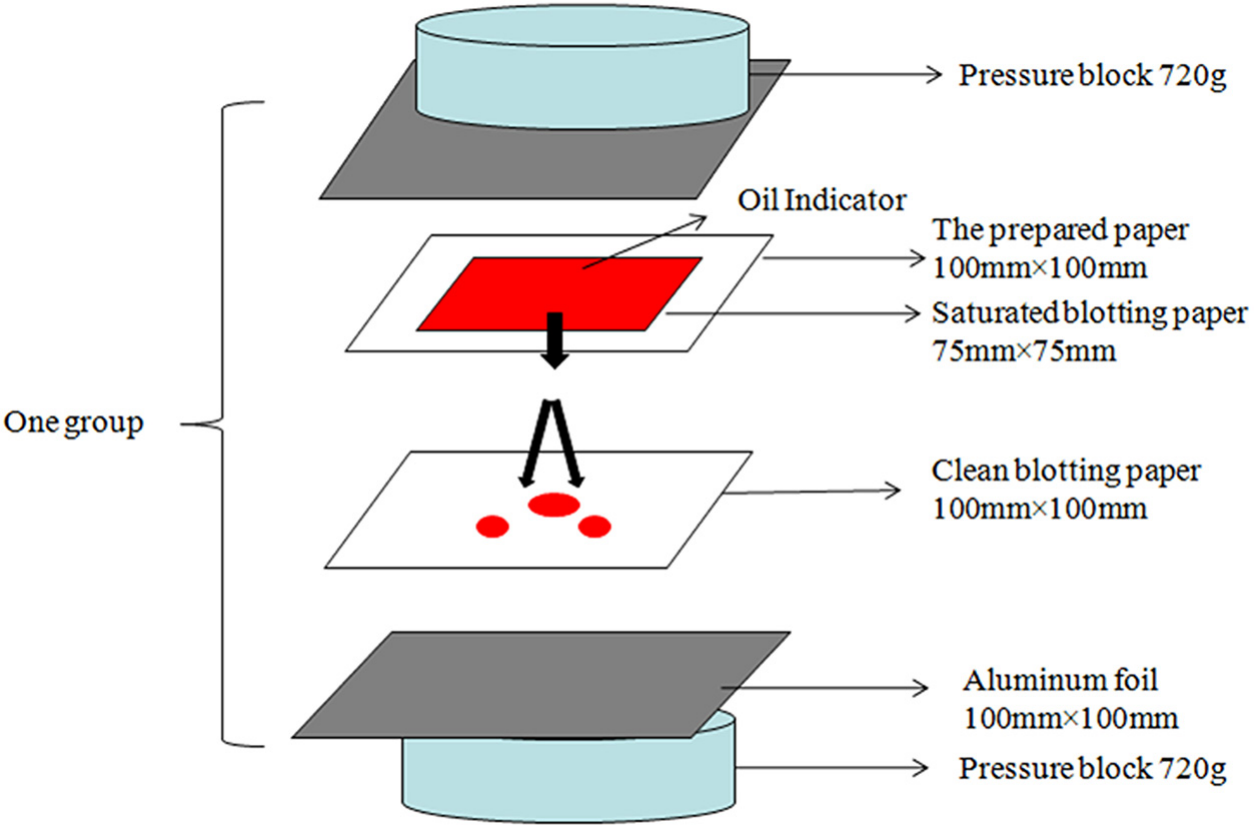
Oil permeability test assembly (TAPPI standards T-507).

The oil resistance was measured according to the modified TAPPI method T-507 cm-99, the procedure of schematic diagram is shown in Fig 2. In this study, vegetable oil was applied for sample testing. The area of the blotters stained with oil was determined by a point–counting method. The results were calculated as the average of five measurements.

#### Scanning electron microscopy and mercury intrusion porosimetry

The surface and cross-sections of the treated paper were observed using scanning electron microscopy (JSM-7600F, JEOL, Japan) operated at 10 kW. The surfaces of the samples were sputter coated with gold prior to observation.

The porosity of the treated paper was measured in accordance with ISO 15901–1:2005 using a mercury intrusion porosimetry (Autopore Ⅳ 9500 Ⅵ.06, micromeritics, USA)

#### Fourier transform infrared spectrum

FT-IR spectra were measured with a FT-IR 360 spectrometer (Thermo Nicolet Corporation, USA) using the ATR-IR method. The IR spectra (4000–400 cm^-1^) were recorded at a resolution of 0.5 cm^-1^ and 40 scans per sample.

#### X-ray diffraction

X-ray diffraction tests were performed at ambient temperature on an X-ray diffractometer (UI tima IV, Japan) using Cu kα radiation at 40 kV and 30 mA. All of the scans were in the range 5°≤2θ≤40°at a step size of 0.05°. The crystallinity was evaluated by Segal’s crystallinity index (CrI), which was calculated using the following equation (Eq 1):

$$CrI=\frac{I-I’}{I}$$ (1)

where I is the diffraction intensity assigned to the (002) plane of cellulose I_β_ and I’ is the intensity measured at 2θ = 18°, which is the maximum in the diffractogram for non-crystalline cellulose [20].

#### Antibacterial assessment of the samples

The inhibition effects of samples with ZnO were measured using the disk diffusion method. *Escherichia coli* (one Gram-negative bacterium) and *Staphylococcus aureus* (one Gram-positive bacterium) were used in the experimentation. The culture medium for microorganisms was a mixture of 15 g of beef extract, 5 g of peptone, 5 g of sodium chloride and 17 g of agar in 1000 ml of water. The pH was regulated to 8.0 by 1 M HCl and 1 M NaOH. A volume of 0.1 ml of bacterial suspension (approximately 10^8^ CFU/ml) was plated and spread on the agar plates before a roundish sheet sample (with a diameter of 15 mm) was placed on the surface of the agar. Then, the dishes were placed an incubator at 37°C for 20 h under light and dark conditions. The antibacterial activity was evaluated by measuring the diameter of the inhibition zones. This process was repeated three times for each sample.

To investigate the morphologic changes of *S*.*aureus* and *E*. *coli* after 24h of treatment using Samples at 37°C, Transmission Electron Microscopy (TEM) (JEM-140) was used. The suspension of *S*. *aureus* and *E*. *coli* were diluted to approximately 1×10^8^CFU/ml before measurement.

#### Analysis of stability and chemical bonding state of the zinc

To examine the release behaviour of the Zn^2+^ ions from the treated paper, the corresponding samples were weighed. Paper samples (1 cm×1 cm) were immersed in vials with 5 ml of distilled water and treated for up to 1 month at 37°C in an orbital shaker at 160 rpm. Then, the samples were removed and the solutions were analysed using flame atomic absorption spectrometry (FAAS) with HCl-HNO_3_ digestion. The content of the ZnO remaining in the treated paper was also evaluated. The sample was burned to ash, and digested with HCl-HNO_3,_ and then measured by FAAS.

The chemical bonding states of the ZnO were identified by X-ray photoelectron spectroscopy (XPS) using an AXIS Ultra DLD system (UK). All of the binding energies were referenced to the C 1s peak at 284.6 eV.

## Results and Discussion

### Mechanical properties

**Fig 3:**
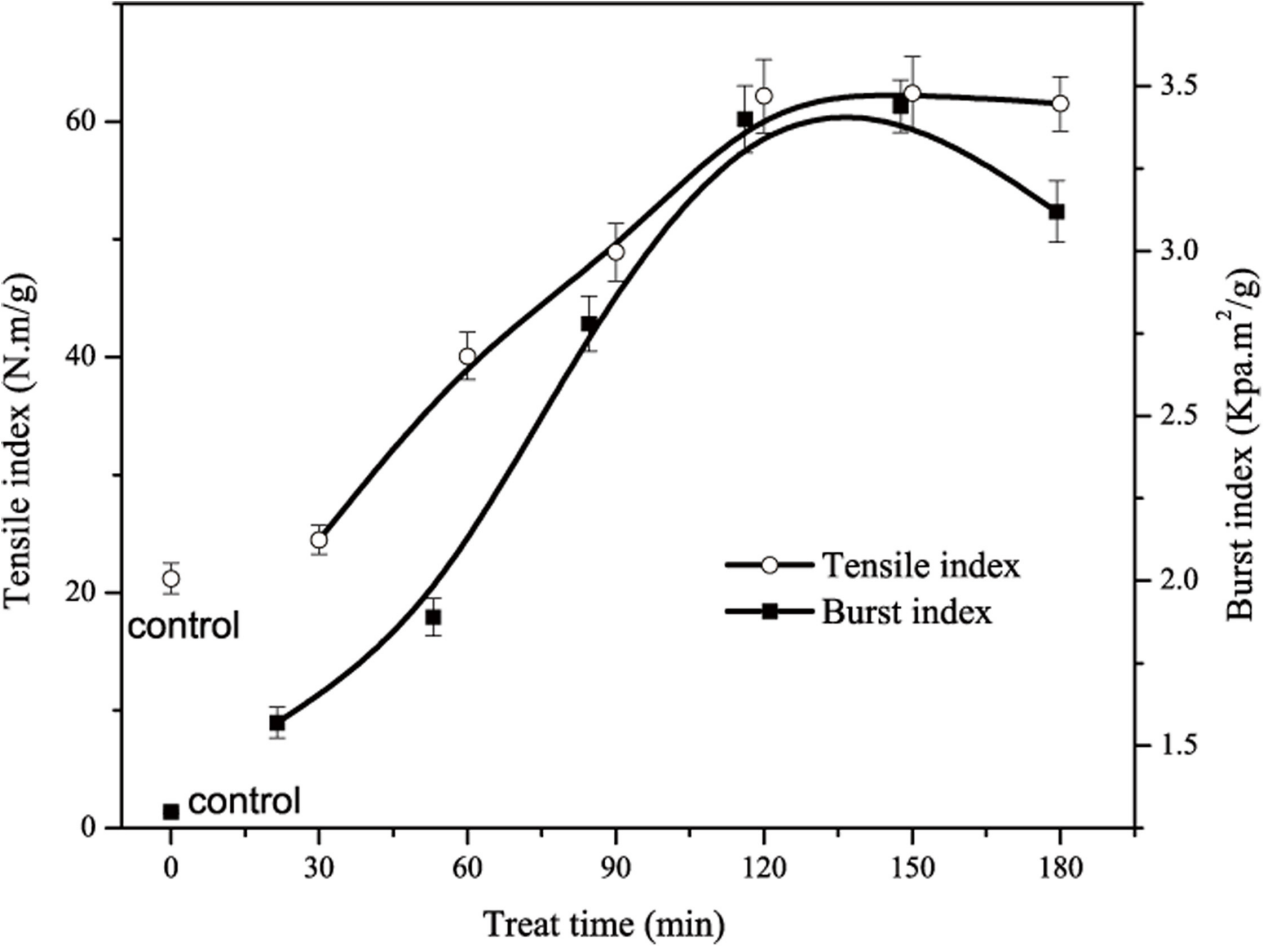
Effect of treat time on mechanical properties of treated paper.

It has been reported that cellulose can be dissolved rapidly in a 7–10 wt.% NaOH/ 12 wt.% urea aqueous solution pre-cooled to -12°C. It cannot be dissolved in the same solvent without prior cooling because the exothermic dissolution reactions of cellulose in alkali solutions prefer a lower temperature. Surface layers of cellulose fibres can be continually dissolved with increasing time at -12°C [21–23]. First, loose amorphous cellulose from the paper sheet were swelled, and hydrogen bonds of amorphous celluloses were destroyed to form dissolved fibres. With continuous swelling, partial crystalline cellulose was also dissolved. After regeneration in water, large numbers of hydrogen bonds were rearranged between the dissolved cellulose and un-dissolved fibres. The well-formed interface bonds allowed for a greater interfacial transfer when the treated paper was mechanically stressed. As shown in Fig 3, the tensile index of the treated paper for 120 min was two times higher than that of the filter paper (the control), and the burst index was 150% higher compared to the control sample. However, more crystalline cellulose were dissolved with continuous penetration, resulting in a reduction in the mechanical properties [24,25].

### Evaluation of oil-resistance

**Fig 4:**
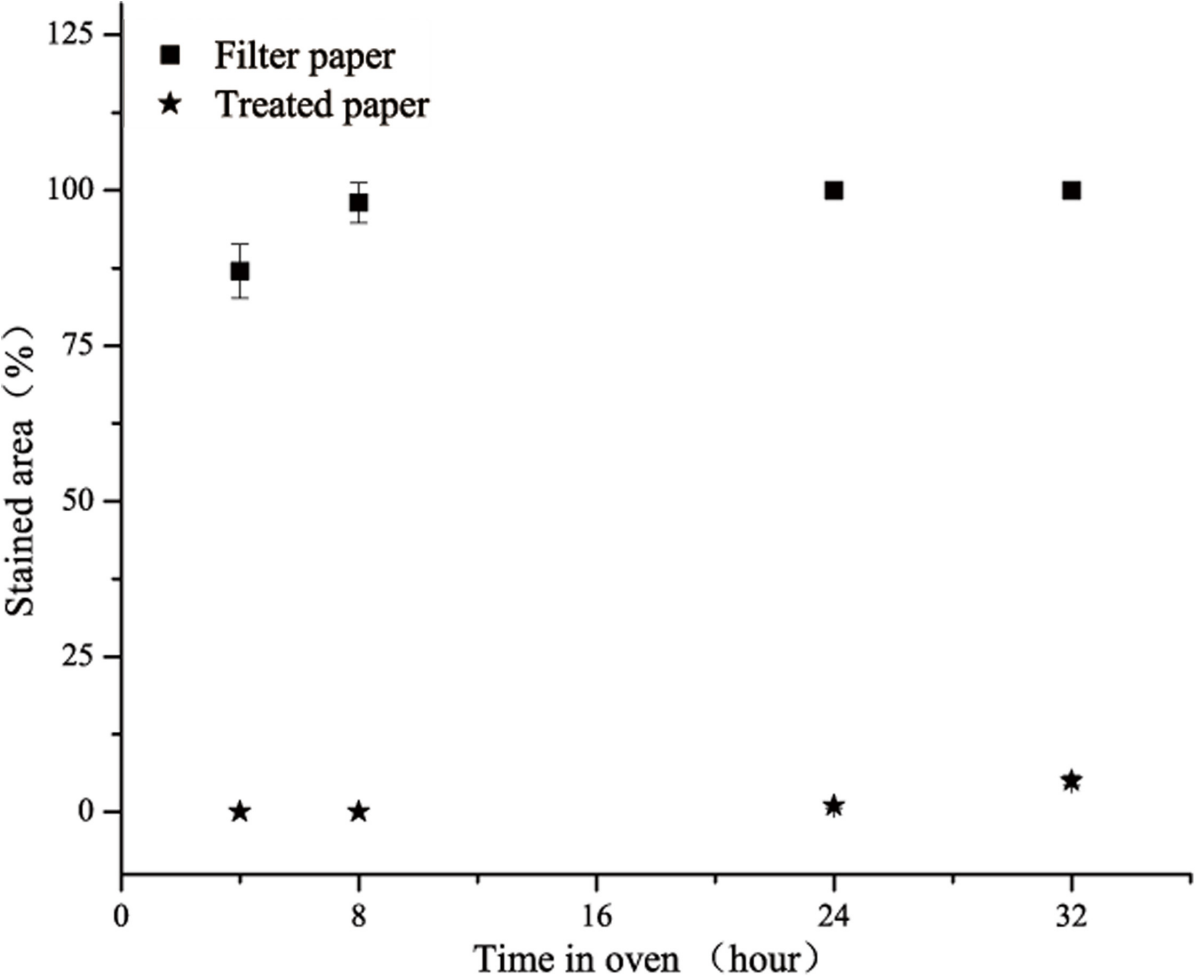
Oil resistance of treated paper-120min compared with filter paper.

Compared to the original filter paper, the treated paper demonstrated a better oil resistance, as shown in Fig 4. The control sample exhibited a 100% strained area at 60°C for 4 h. However, the treated paper only exhibited a 5% strained area, even at 60° for 32 h, resulting in an excellent ability to prevent oil permeation. The oil-resistance of the treated paper-120 min meets the requirements for fast food packaging. The oil-resistance arises from the relative absence of pores in the paper network, which is primarily determined by the largest pore size in the paper. The density structure can resist oil permeation through capillaries [26]. The larger pore size, the more easily oil passes though the network of paper. There was no strained area on treated paper-120 min over 24 h, and the treated paper-120 min shows excellent oil resistance property.

### Morphology and characterization of the paper

**Fig 5:**
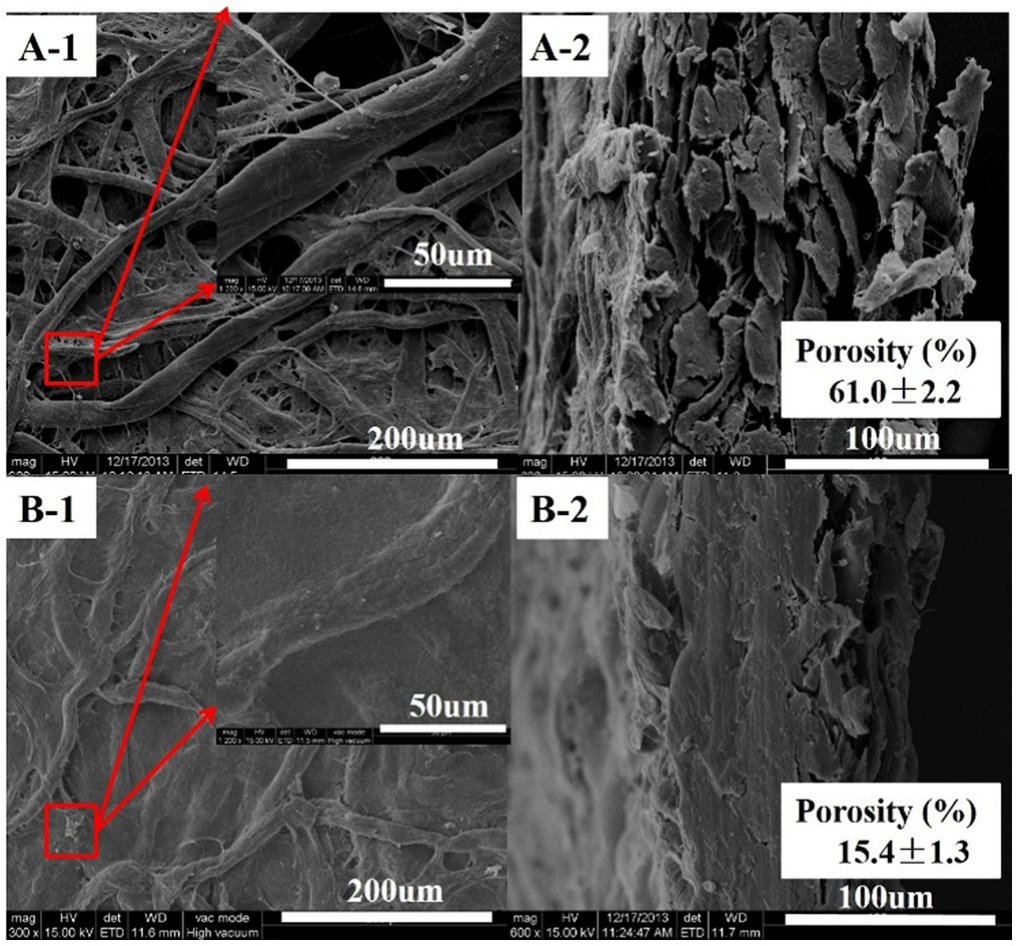
SEM images of filter paper and treated paper-120min (A-1, A-2 are images of the surface and cross-section for treated paper-120min; B-1, B-2 are images of the surface and cross-section for the filter paper.

To investigate the reasons for the enhancement of the mechanical properties and oil-resistance for the treated paper-120 min, scanning electron microscopy (SEM) and mercury intrusion porosimetry (MIP) were used to evaluate the changes in the paper structure and porosity. A fine web-like and high porous network structure was observed in Fig 5A-1 and 5A-2. The treated paper-120 min surface exhibited less porosity and a high level of homogeneity in Fig 5B-1. Additionally, the treated paper-120 min exhibited a compact cross-section, as shown in Fig 5B-2. These results implied a loose amorphous and partially crystalline region of the paper cellulose were dissolved in the NaOH/urea/ZnO aqueous solution and then filled into the pores and covered the un-dissolved fibres. Subsequently, more hydrogen bonds were formed during regeneration in water. The partially dissolved fibres acted as a glue to join the un-dissolved fibres and formed a high quality interface with the same cellulose, reducing the appearance of interfacial tension between the two different components. It also allowed more stress transfer from fibre to fibre, leading to greater strength and less porosity. These results were confirmed by MIP analysis, as shown in Fig 5. The porosity of the filter paper decreases from 61.0% to 15.4%, leading to the excellent oil-resistance and high mechanical properties of the treated paper-120 min.

**Fig 6:**
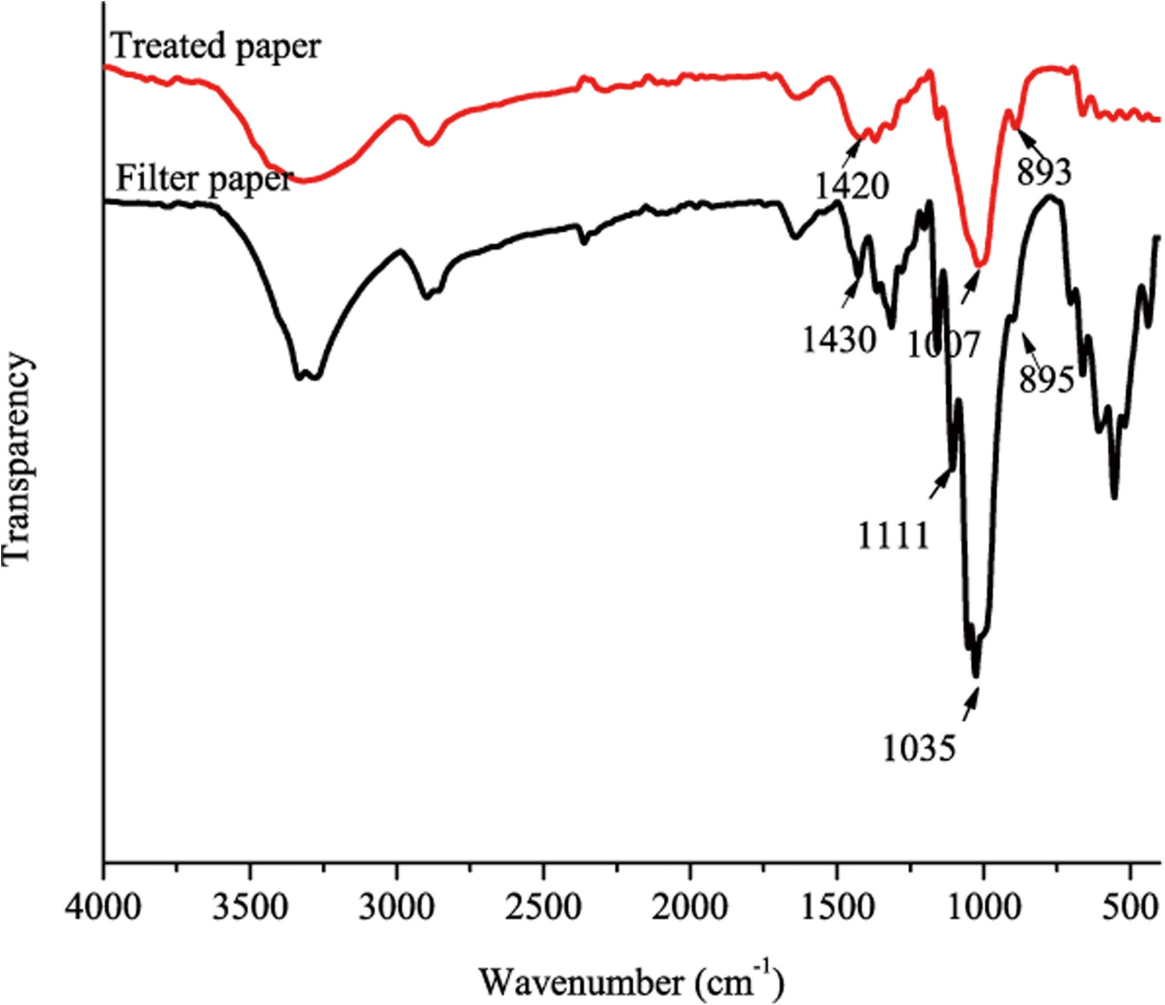
FT-IR spectra of treated paper-120min compared with filter paper.

The structural changes in the treated paper-120 min compared to the filter paper were also characterized. As shown in Fig 6, the treated paper-120 min and filter paper exhibited characteristic peaks at approximately 3400 cm^-1^, which were assigned to–OH stretching intra-molecular hydrogen bonds. In addition, the peak at 3400 cm^-1^ from the treated paper-120 min was obviously broadened and shifted to a lower wavelength, suggesting an increase in the inter-molecular hydrogen bonding with the cellulose [27]. During the cellulose dissolution process, the amorphous and partially crystalline regions were dissolved, and their intra- and inter-molecular hydrogen bonds were broken. After regeneration in distilled water, more hydrogen bonds were uniformly rearranged [28]. Furthermore, several characteristic bands were obviously shifted at the peak maximum or the absorbance changed. After treatment with 8%NaOH/12%urea/0.25%ZnO, the bands of cellulose at 1430, 1111 and 895 cm^-1^ were shifted to 1420, 1007 and 893 cm^-1^, respectively. These are typical changes relating to cellulose crystalline transformation (Ⅰ to Ⅱ). Shifting to 1420 cm^-1^ suggested the formation of new inter- and intra-molecular hydrogen bonds and a change for the CH_2_OH at C-6 from the tg to the gt form. The content of the cellulose Ⅱ is more significant, and the band at 1420 cm^-1^ will be widened [29]. The absorbing peak at 1111 cm^-1^ shifted to 1007 cm^-1^ and was assigned to ring asymmetric stretching. The absorption band at 895 cm^-1^ shifted to 893 cm^-1^ and was assigned to C-O-C stretching at the β-(1–4)-glycosidic linkage and corroborated the near total absence of crystalline cellulose Ⅰ. The absorbances at 1430, 1111 and 895 cm^-1^ are sensitive to the ratio of crystalline to amorphous structure in the cellulose, and the broadening of these bands indicates a more disordered structure [30,31]. Therefore, the analysis of the FT-IR spectrum indicated that the amount of crystalline region decreased and the amount of amorphous region was increased. Subsequently, more inter-molecular hydrogen bonds were formed.

**Fig 7:**
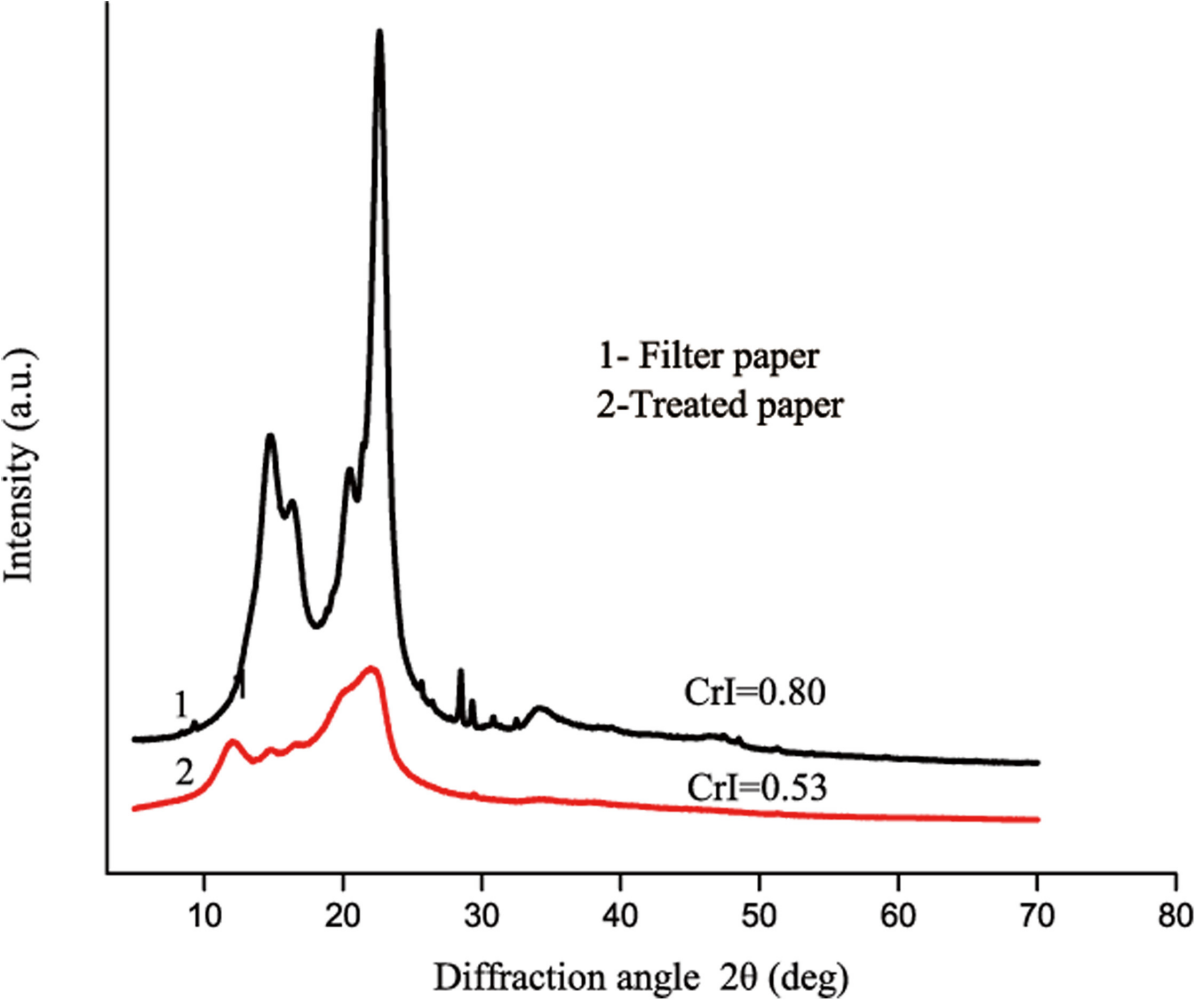
X-ray diffraction (X-RD) patterns of treated paper compared with filter paper.

Fig 7 shows the XRD patterns of the treated paper-120 min and the filter paper. The filter paper exhibited characteristic absorptions for cellulose Ⅰ at 2θ = 14.7° for the (101) plane, 2θ = 16.6° for the (10–1) plane, and 2θ = 22.7° for the (002) plane. The peaks of the prepared samples at 2θ = 12.3°, 20.3°and 22.0° were characteristic diffractions of cellulose Ⅱ crystals and the (1–10), (110) and (200) planes, implying the transformation of cellulose crystals from Ⅰ to Ⅱ [32]. The amount of CrI (0.80) in the filter paper dropped to 0.53 after treatment, which suggested that partial crystalline cellulose dissolved in 8%NaOH/12%urea/0.25%ZnO system. This is consistent with the FT-IR results.

### Antibacterial properties

**Fig 8:**
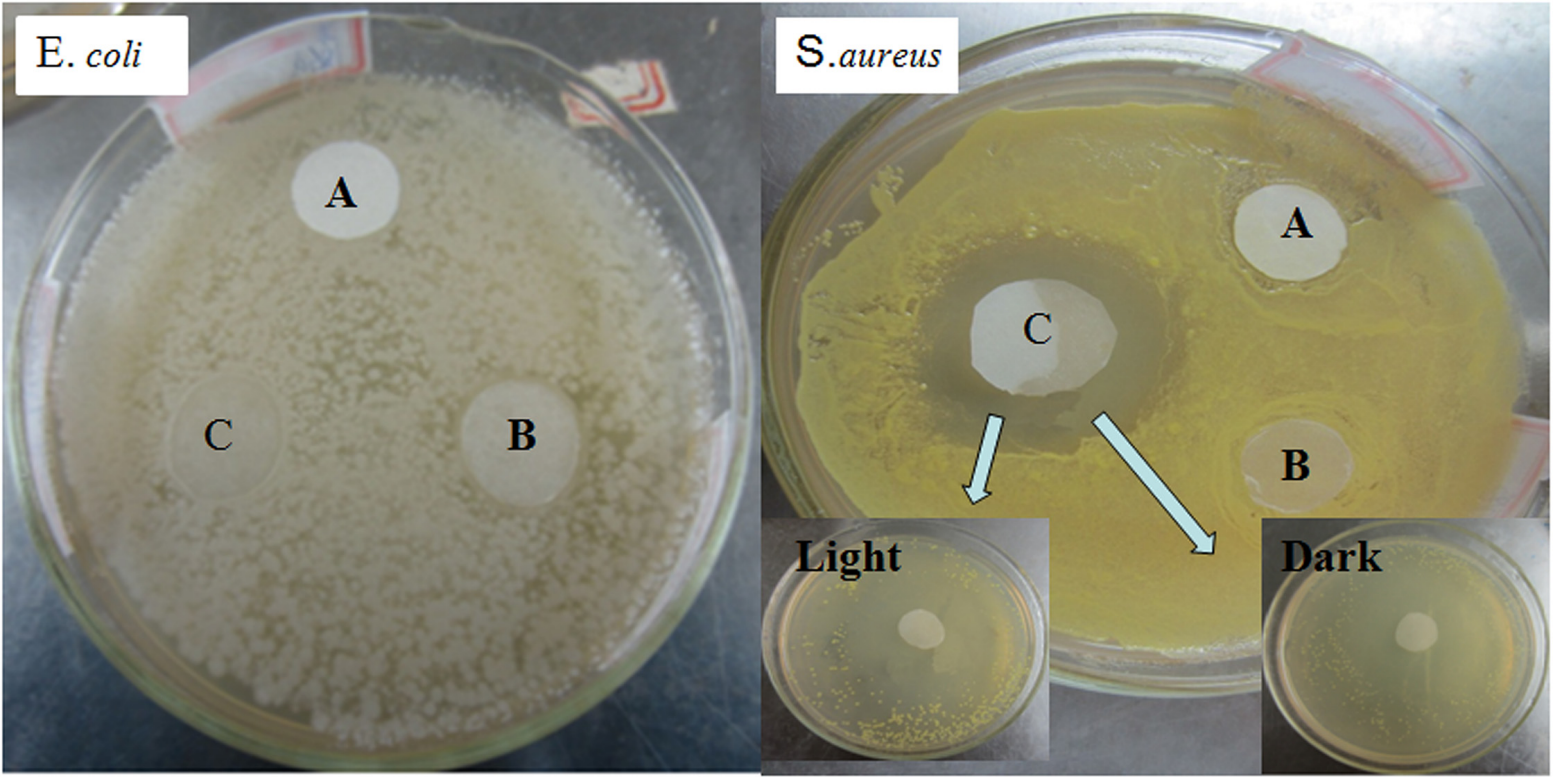
The antibacterial properties of treated paper for *E*.*coli* and *S*. *aureus* (A -filter paper, B-filter paper treated with 8wt%NaOH/12wt%urea aqueous solution in -12°C 120min, C- treated paper-120min).

**Fig 9:**
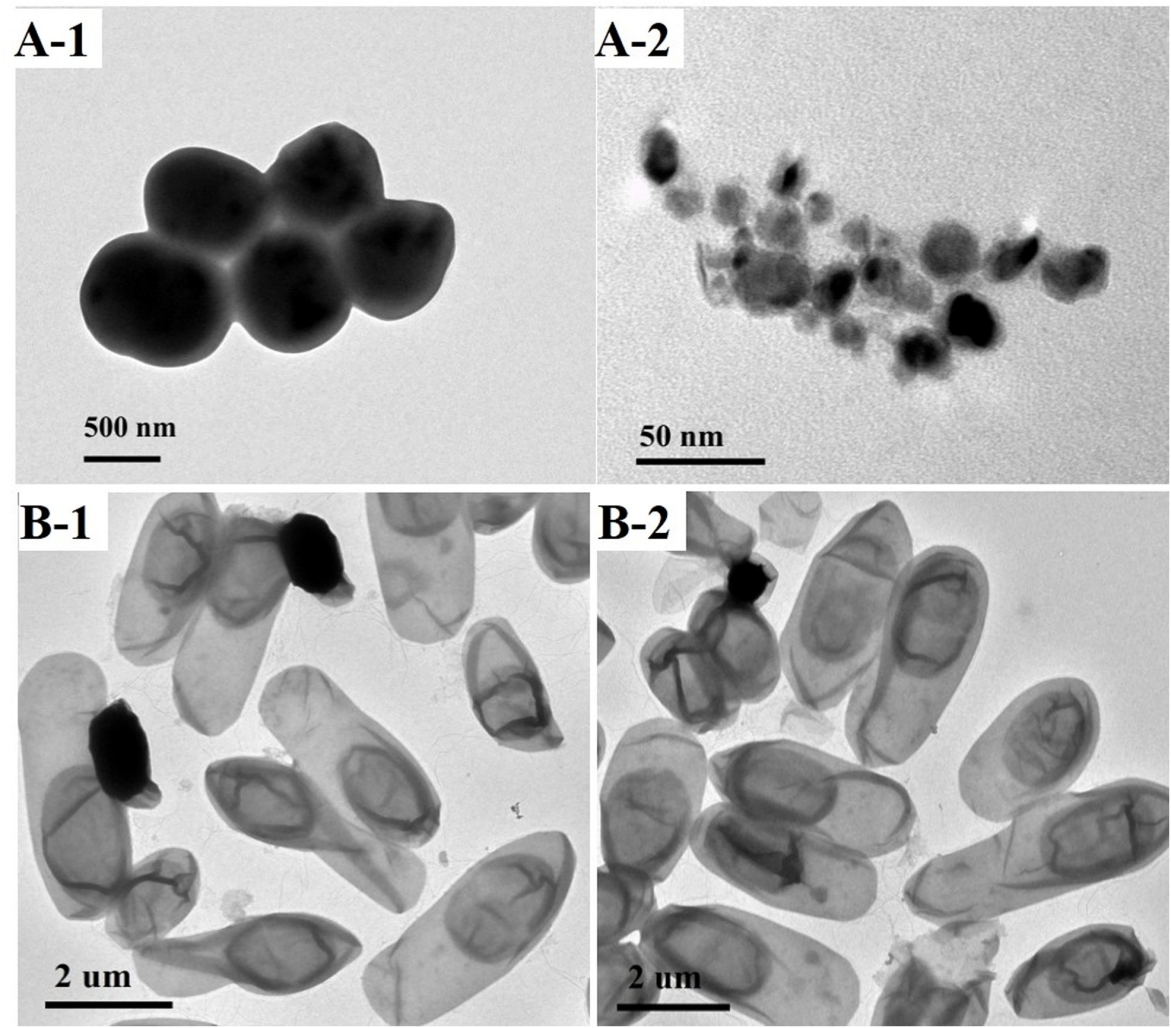
TEM images of *S*. *aureus* and *E*. *coli*. A-1 *S. aureus* untreated cells. A-2: *S. aureus* treated by samples containing ZnO. B-1: E. coli untreated cells. B-2: *S. aureus* treated by samples containing ZnO.

Zinc oxide (ZnO) existed as Zn(OH)_4_^2-^ when added to the NaOH/urea aqueous solution. Zn(OH)_4_^2-^ was converted to ZnO again during the rinsing and drying processes and remained in the paper sheet. ZnO is an efficient antimicrobial agent for a broad range of bacteria targets. It can effectively kill Gram positive and Gram negative bacteria at an appropriate dosage [33,34]. As shown in Fig 8, there was a similar zone of inhibition against *S*. *aureus* around the treated paper sample for both light and dark conditions. However, there was no apparent inhibition zone around the *E*. *coli* sample. Ameer Azam reported that Gram-negative bacterial strains of *E*. *coli* possessed inhibition-zone sizes smaller than Gram-positive bacterial strains of *S*. *aureus* for ZnO nanoparticles [35]. This indicated that the *E*. *coli* strain exhibited a higher resistance/tolerance against ZnO than the *S*. *aureus* strain. Aqueous suspensions containing 4.45×10^−5^–1.25×10^−3^ M ZnO particles exhibit a strong antibacterial activity against *E*. *coli* in dark conditions [36]. In this study, there was no apparent inhibition-zone for the *E*. *coli* strain, which was ascribed to less residual ZnO in the paper and *S*. *aureus* being less resistance than *E*. *coli* because of different cell wall components. Fig 9 shows the apparent morphologic changes of *S*. *aureus* after treatment of Samples. The shape size of *S*. *aureus* decreased from 0.8um to 0.02um or less. However the morphology of *E*.*coli* didn’t show obvious changes. These are in accordance with results of inhibition zone.

There are three main mechanisms reported by researchers: (1) ZnO damages the structure of the microbial cell membrane and the internal components of cell, causing cytoplasm leakage and the death of bacterial cells [33,37,38], (2) Zinc ion release leads to the inhibition of multiple activities in the bacteria, such as glycolysis transmembrane proton translocation and acid tolerance [39], (3)and the generation of reactive oxygen species (ROS) by photolytic or non-photolytic, which causes fatal damage to cellular constituents [39–42]. The amount of zinc released from the treated paper and its residual content in the paper were evaluated. AAS spectra did not show any absorption of ZnO, implying that ZnO or Zn^2+^ released from the treated paper is not significant or escapes the detection limit of this analysis. ZnO was stably fixed on the treated paper, and Zn^2+^ did not contribute to any antibacterial effect. Many studies have indicated that the formation of ROS is the main antibacterial mechanism of ZnO [43–46]. Many studies have clearly indicated that ZnO nanoparticle or powders can produce ROS such as hydroxyl radical, superoxide anion and hydrogen peroxide [47,48]. Electron-hole pairs would be generated when ZnO is activated by light. Subsequently, the electron-hole may combine with H_2_O to produce OH^-^ and H^+^. Oxygen molecules absorbed electrons released from ZnO and turned them into superoxide radical anions (•O_2_^-^), which in turn reacted with H^+^ to generate HO_2_• radicals. Finally HO_2_• combined with hydrogen ions and electrons to produce H_2_O_2_ [49]. H_2_O_2_ can penetrate into bacteria cells and cause death. Without photocatalysis, ZnO can react with H_2_O to produce HO• before producing H_2_O_2_ by the combination of two HO•. Therefore, ZnO can exhibit antibacterial property under light or in dark [50]. Dutta et al. reported that production of ROS is the key phenomenon for antibacterial effect of ZnO nanoparticles. The generated ROS in the culture media was capable to cause oxidation of lip membrane in the cell wall [44]. Lakshmi et al. studied the mechanism of antibacterial activity of ZnO. They also found the mechanism of antibacterial activity of ZnO is attributed mainly to ROS even in the dark [46]. Some other researchers find the same results about the mechanism of antibacterial activity of ZnO [51–53]. ROS plays an important role in antibacterial activity of ZnO.

**Fig 10:**
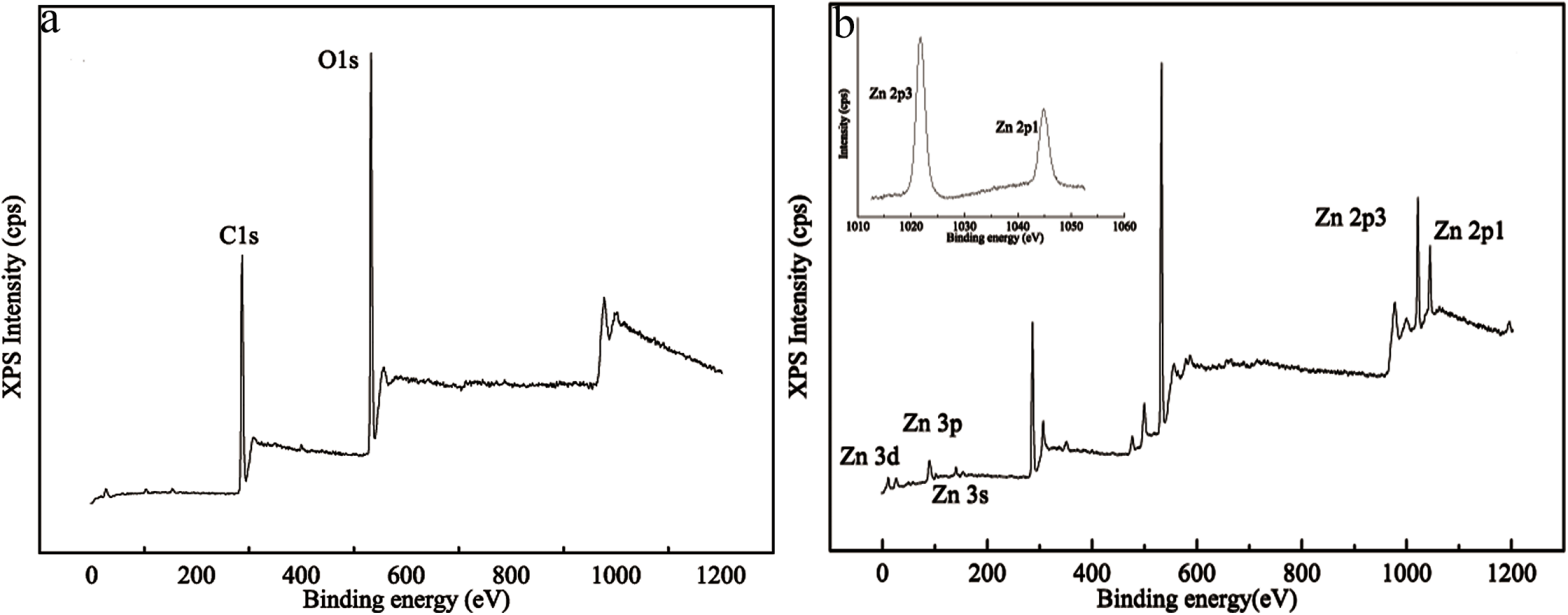
XPS spectrum of the filter paper (a), the filter paper treated by NaOH/urea/ZnO solution (b).

XPS analysis was performed to identify the presence of ZnO. In comparison with Fig 10(A), in Fig 10(B) there are not only C1s and O1s peaks, peaks corresponding to Zn 2p, Zn 3s, Zn 3p and Zn 3d also emerged in the XPS spectra of the paper treated by 8 wt. % NaOH/12 wt. % urea/0.25 wt. % ZnO solution, indicating the modified paper was mainly composed of zinc, oxygen and carbon. The binding energies of Zn 2p3 and Zn 2p1 was determined to be 1022.3 and 1045.4 eV, and the peak separation between them was 23.1 eV. According to the results, the zinc ions were mainly in the form of ZnO [54–56].

The ZnO remaining in the treated paper-120 min was 4.63 μg per 1 g of cellulose, as was determined from FAAS analysis. This is a small amount, which is in the testing range for the FT-IR and XRD, as shown in Fig 6 and Fig 7. There was no characteristic peak for ZnO. COLIPA reported that the oral half lethal dose of ZnO for a mouse is greater than 2000 mg/kg [57]. The ZnO retained in the treated paper is much less than half the lethal dose. Therefore, treated paper is safe for use as a package material and other functional substrate.

## Conclusions

A new self-reinforced antibacterial paper with oil resistance was successfully prepared using a NaOH/Urea/ZnO system. The surfaces of the cellulose were dissolved, and pores were filled between fibres and coated on un-dissolved highly oriented fibre cores, which effectively reduced the porosity of the paper. This assembly imparted an effective reinforcement effect to the paper. During the 120-min treatment, interfacial adhesion between the dissolved and un-dissolved cellulose allowed more stress transfer capabilities in this treated paper. Mechanical testing showed that the tensile and burst indexes were approximately three times higher compared to the filter paper. In addition, the treated paper exhibited total oil-resistance abilities with reducing the porosity for 24 h. The trace fixed ZnO in the treated paper gave the modified paper an excellent antibacterial property for S. aureus. The functional paper is capable of being utilized in packaging and other fields due to its outstanding properties.
